# Is Long-Term Low-Dose Aspirin Therapy Associated with Renal Dysfunction in Patients with Type 2 Diabetes? JPAD2 Cohort Study

**DOI:** 10.1371/journal.pone.0147635

**Published:** 2016-01-25

**Authors:** Sadanori Okada, Takeshi Morimoto, Hisao Ogawa, Mio Sakuma, Hirofumi Soejima, Masafumi Nakayama, Hideaki Jinnouchi, Masako Waki, Yasuhiro Akai, Hitoshi Ishii, Yoshihiko Saito, investigators for the Japanese Primary Prevention of Atherosclerosis with Aspirin for Diabetes (JPAD) trial

**Affiliations:** 1 Department of Diabetology, Nara Medical University, Kashihara, Nara, Japan; 2 First Department of Internal Medicine, Nara Medical University, Kashihara, Nara, Japan; 3 Department of Clinical Epidemiology, Hyogo College of Medicine, Nishinomiya, Hyogo, Japan; 4 Department of Cardiovascular Medicine, Graduate School of Medical Science, Kumamoto University, Kumamoto, Japan; 5 Diabetes Care Center, Jinnouchi Hospital, Kumamoto, Japan; 6 Division of Endocrinology and Metabolism, Department of Internal Medicine, Shizuoka City Hospital, Shizuoka, Japan; 7 Department of Regulatory Medicine of Blood Pressure, Nara Medical University, Kashihara, Nara, Japan; Ichan School of Medicine at Mount Sinai, UNITED STATES

## Abstract

**Background:**

Low-dose aspirin is widely recommended for patients at high risk for cardiovascular disease (CVD); however, it remains uncertain whether long-term treatment adversely affects renal function in patients with diabetes.

We investigated whether long-term low-dose aspirin affects renal dysfunction in patients with diabetes.

**Methods:**

We conducted a randomized controlled trial (RCT), the Japanese Primary Prevention of Atherosclerosis with Aspirin for Diabetes (JPAD) trial, to evaluate low-dose aspirin as primary prevention for CVD in patients with type 2 diabetes. We followed the patients with negative urine dipstick albumin of the JPAD trial in a cohort study after the RCT period was completed. Patients were randomly allocated to receive aspirin (81 mg or 100 mg daily, aspirin group) or no aspirin (no aspirin group). After the RCT, the treating physician decided whether to administer aspirin. We evaluated the incidence of positive urine dipstick albumin and annual changes in estimated glomerular filtration rate (eGFR).

**Results:**

Positive urine dipstick albumin developed in 297 patients in the aspirin group (n = 1,075) and 270 patients in the no aspirin group (n = 1,098) during follow-up (median, 8.5 years). Intention-to-treat analysis showed low-dose aspirin did not increase the incidence of positive urine dipstick albumin (hazard ratio [HR], 1.17; 95% confidence interval [CI], 0.995–1.38). On-treatment analysis yielded similar results (HR, 1.08; 95% CI, 0.92–1.28). Multivariable analysis showed the incidence of positive urine dipstick albumin was higher among the elderly and those with elevated serum creatinine, high hemoglobin A1c, or high blood pressure; however, low-dose aspirin did not increase the risk of positive urine dipstick albumin. There were no significant differences in annual changes in eGFR between the groups (aspirin, −0.8 ± 2.9; no aspirin, −0.9 ± 2.5 ml/min/1.73m^2^/year).

**Conclusion:**

Long-term low-dose aspirin does not affect eGFR and positive urine dipstick albumin in patients with type 2 diabetes.

## Introduction

Low-dose aspirin therapy is widely recommended for preventing cardiovascular disease (CVD) [1]. Individuals who are at high risk for CVD may take low-dose aspirin for life. However, it remains uncertain whether long-term treatment is safe with respect to renal function because aspirin belongs to a class of cyclooxygenase inhibitors. The inhibition of cyclooxygenase decreases production of prostaglandin in the kidney, and reduces renal blood flow and glomerular filtration rate. In patients with reduced renal function, this may result in retention of water, hypertension and, in some case, to renal failure [2]. Previous clinical studies have yielded conflicting results about aspirin and the risk of chronic kidney disease (CKD) or end-stage renal disease (ESRD) [3–13]. There have been very few studies on the relationship between aspirin and CKD in patients with diabetes.

Low-dose aspirin therapy is also recommended for CVD prevention in high-risk diabetic patients with a history of CVD [14] or CVD risk factors [15]. Patients with diabetes are at high risk not only for CVD, but also CKD secondary to diabetic nephropathy [16, 17]. Albuminuria or proteinuria occur early in the course of diabetic nephropathy, before an appreciable decline in the estimated glomerular filtration rate (eGFR) [18]. However, renal function in previous studies was evaluated based on serum creatinine levels, creatinine clearance, or eGFR, not the presence of albuminuria or proteinuria. Therefore, we thought it was necessary to assess the long-term risk of low-dose aspirin therapy based on the incidence of albuminuria or proteinuria in patients with diabetes.

The Japanese Primary Prevention of Atherosclerosis with Aspirin for Diabetes (JPAD) trial was a randomized controlled trial (RCT) evaluating whether low-dose aspirin prevents CVD in patients with type 2 diabetes and no history of CVD [19]. We followed the patients of the JPAD trial in a cohort study (JPAD2 cohort study) after the RCT was completed. In the JPAD2 cohort study, we analyzed whether long-term low-dose aspirin therapy affects eGFR and the incidence of positive urine dipstick albumin in patients with type 2 diabetes.

## Methods

The original JPAD trial was a multicenter, prospective, randomized, open-label, blinded end-point trial conducted at 163 institutions throughout Japan. This trial was performed according to the Declaration of Helsinki and was approved by the ethics committee of each participating hospital (Nara Medical University Ethics Committee and Graduate School of Medical Science, Kumamoto University Ethics Committee). Written informed consent was obtained from each participant before participation in the original JPAD trial. In the JPAD2 cohort study, the revised study protocol was approved by the ethics committees (Nara Medical University Ethics Committee and Graduate School of Medical Science, Kumamoto University Ethics Committee). Verbal informed consent was obtained at the start of the JPAD2 cohort study, according to the approval by the ethics committees. The study protocol of the JPAD trial was registered at clinicaltrials.gov with the identifier NCT00110448.

**Fig 1:**
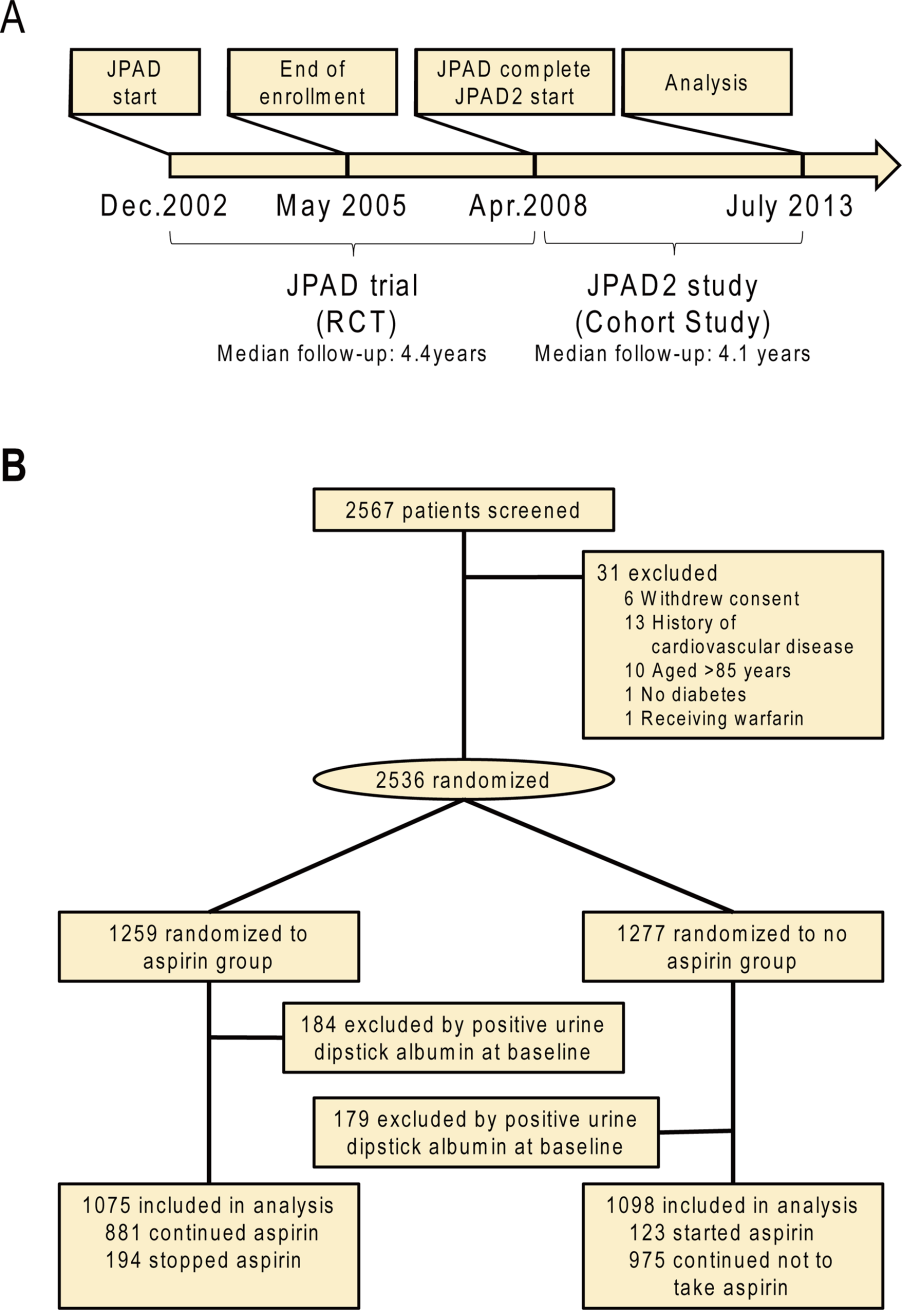
Timeline of the JPAD2 cohort study. **A,** The timeline of the JPAD trial and the JPAD2 cohort study. RCT indicates randomized controlled trial. **B,** The flow chart of the JPAD trial and the JPAD2 cohort study.

Details on the design of the JPAD trial have been previously described [19]. In brief, patient enrollment started in December 2002 and was completed in May 2005. We enrolled 2,536 Japanese patients with type 2 diabetes between 30 and 85 years of age without a history of CVD, who were randomly allocated to receive aspirin (81 mg or 100 mg daily, aspirin group) or no aspirin (no aspirin group). All patients were allowed to undergo all concurrent treatments. After the JPAD trial was completed in May 2008, we started the JPAD2 cohort study. In the JPAD2 cohort study, the decision on whether to administer low-dose aspirin depended on the treating physician’s clinical judgment (Fig 1).

In the present analysis, we defined newly developed positive urine dipstick albumin as the primary endpoint. The positive urine dipstick albumin was defined as ‘1+’ and over. We assessed the annual incidence of positive urine dipstick albumin to July 2013. We followed patients with negative urine dipstick albumin at baseline of the original JPAD trial, and assessed the effect of low-dose aspirin on the incidence of positive urine dipstick albumin.

We also evaluated annual changes in eGFR in each group. eGFR (ml/min/1.73 m^2^ of body surface area) was calculated using the new three-variable Japanese equation for estimating glomerular filtration rate: eGFR = 194 x serum creatinine^-1.094^ x age^-0.287^ x 0.739 (if female) [20]. Annual changes in eGFR were calculated as eGFR divided by time between baseline and follow-up (years). We analyzed differences in the annual change in eGFR between the aspirin and no aspirin groups. Patients whose follow-up period was less than 1 year were excluded from the analysis.

### Statistical analyses

Categorical variables were expressed as numbers and percentages. Continuous variables were expressed as means ± SD or medians (interquartile range [IQR]). Based on their distribution, continuous variables were compared using the Student’s t-test or Wilcoxon rank sum test as appropriate. Efficacy comparisons were made on the basis of time to the first event according to the intention-to-treat principle. Patients lost to follow-up were censored on the day of their last visit. We used Cox proportional hazards models to estimate hazard ratios (HRs) and 95% confidence intervals (CIs). The cumulative incidence of each endpoint was estimated using the Kaplan-Meier method in each group, and differences between groups were assessed with the log-rank test. Next, we performed an on-treatment analysis, based on whether patients were actually taking aspirin during the follow-up period, using Cox proportional hazards modeling and the Kaplan-Meier method.

We developed multivariable Cox proportional hazards models to assess the effect of aspirin on the incidence of positive urine dipstick albumin in both intention-to-treat and on-treatment analyses. We employed the following variables in the multivariable models: age (≥65 years at baseline), sex, baseline serum creatinine level (continuous variable), hemoglobin A1c (HbA1c) (binary variable divided by a median value), blood pressure (systolic blood pressure ≥140 mmHg or diastolic blood pressure ≥90 mmHg), and treatment with angiotensin-converting enzyme (ACE) inhibitors or angiotensin II type 1 receptor blockers (ARBs). The mean HbA1c and blood pressure values at baseline and each annual follow-up were used. Treatment with ACE inhibitors or ARBs was found to be associated with renal function in previous studies [21, 22].

Statistical analyses were conducted by an independent statistician (T.M.) with the use of JMP 8.0 (SAS Institute, Cary, NC) and SAS 9.4 (SAS Institute) software. *P* values less than 0.05 were considered statistically significant.

## Results

### Patient characteristics at baseline

We included 2,173 patients with negative urine dipstick albumin at baseline of the original JPAD trial (Table 1). There were 1,075 patients in the aspirin group and 1,098 patients in the no aspirin group. The mean age ± SD was 65 ± 10 years in the aspirin group and 64 ± 10 years in the no aspirin group (*P* = 0.01). The aspirin group had a slightly higher mean serum creatinine level than the no aspirin group (aspirin group, 68 ± 19 μmol/L; no aspirin group, 66 ± 18 μmol/L; *P* = 0.04), but there were no significant differences in eGFR at baseline (aspirin group, 74.0 ± 19.3 ml/min/1.73m^2^; no aspirin group, 75.6 ± 20.0 ml/min/1.73m^2^; *P* = 0.06). Diastolic blood pressure was significantly higher by 1 mmHg in the aspirin group (aspirin group, 77 ± 9 mmHg; no aspirin group, 76 ± 9; *P* = 0.03). There were no differences in systolic blood pressure, prevalence of hypertension, and use of antihypertensives.

**Table 1:** Patients’ characteristics at baseline of the original JPAD trial.

	Aspirin	No aspirin	*P* value
N	1075	1098	
Age, y	65 ± 10	64 ± 10	0.01
Male	592 (55)	573 (52)	0.2
BMI, kg/m^2^	24 ± 4	24 ± 4	0.4
Duration of diabetes, y	7.2 (2.8–12.2)	6.5 (2.9–12.1)	0.4
Hypertension	604 (56)	605 (55)	0.6
Dyslipidemia	578 (54)	568 (52)	0.3
History of smoking	234 (22)	210 (19)	0.1
Systolic BP, mmHg	135 ± 15	134 ± 14	0.06
Diastolic BP, mmHg	77 ± 9	76 ± 9	0.03
FPG, mmol/L	8.1 ± 2.7	8.0 ± 2.6	0.7
HbA1c, %	7.5 ± 1.4	7.4 ± 1.2	0.09
Serum creatinine, μmol/L	0.77 ± 0.22	0.75 ± 0.20	0.04
eGFR, ml/min/1.73m^2^	74.0 ± 19.3	75.6 ± 20.0	0.06
Total cholesterol, mmol/L	5.22 ± 0.88	5.17 ± 0.88	0.1
Fasting triglycerides, mmol/L	1.28 (0.89–1.78)	1.25 (0.89–1.81)	0.5
HDL cholesterol, mmol/L	1.45 ± 0.41	1.45 ± 0.39	0.9
Medications			
Sulfonylurea	619 (58)	604 (55)	0.2
α-glycosidase inhibitor	353 (33)	363 (33)	0.9
Biguanide	134 (12)	156 (14)	0.2
Thiazolidinedione	51 (5)	49 (4)	0.8
Insulin	138 (13)	130 (12)	0.5
Calcium channel blocker	344 (32)	358 (33)	0.8
ACE inhibitor	145 (13)	159 (14)	0.5
ARB	209 (19)	210 (19)	0.9
β-blocker	61 (6)	76 (7)	0.2
Statin	266 (25)	292 (27)	0.3

Duration of diabetes and fasting triglyceride levels are expressed as medians (interquartile range). BMI indicates body mass index; BP, blood pressure; FPG, fasting plasma glucose; eGFR, estimated glomerular filtration rate; HDL, high- density lipoprotein; ACE, angiotensin-converting enzyme; and ARB, angiotensin II type 1 receptor blocker.

### Effect of low-dose aspirin on the incidence of positive urine dipstick albumin

**Fig 2:**
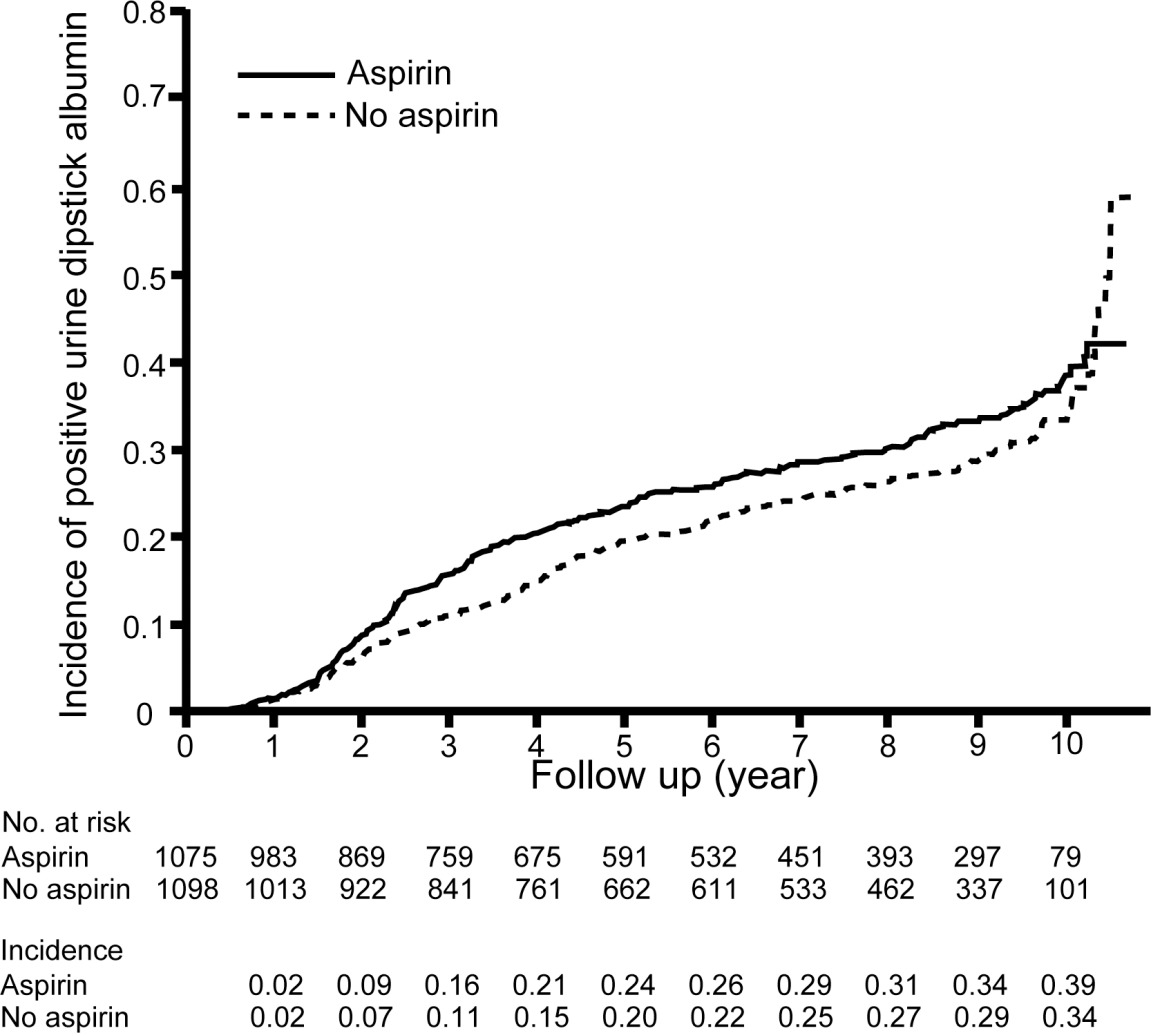
Incidence of positive urine dipstick albumin in patients on long-term low-dose aspirin therapy in the intention-to-treat analysis. Positive urine dipstick albumin developed in 297 patients in the aspirin group and 270 patients in the no aspirin group. The intention-to-treat analysis showed that low-dose aspirin did not increase the incidence of positive urine dipstick albumin (HR, 1.17; 95% CI, 0.995 to 1.38; log-rank *P* = 0.057)

The median duration of follow-up was 8.5 years (95% CI, 8.3 to 8.7 years), which includes the RCT period of the JPAD trial. During the follow-up period, positive urine dipstick albumin developed in 297 patients in the aspirin group and 270 patients in the no aspirin group. Kaplan-Meier curves based on the intention-to-treat principle showed that low-dose aspirin does not increase the incidence of positive urine dipstick albumin (HR, 1.17; 95% CI, 0.995 to 1.38; log-rank *P* = 0.057; Fig 2).

**Fig 3:**
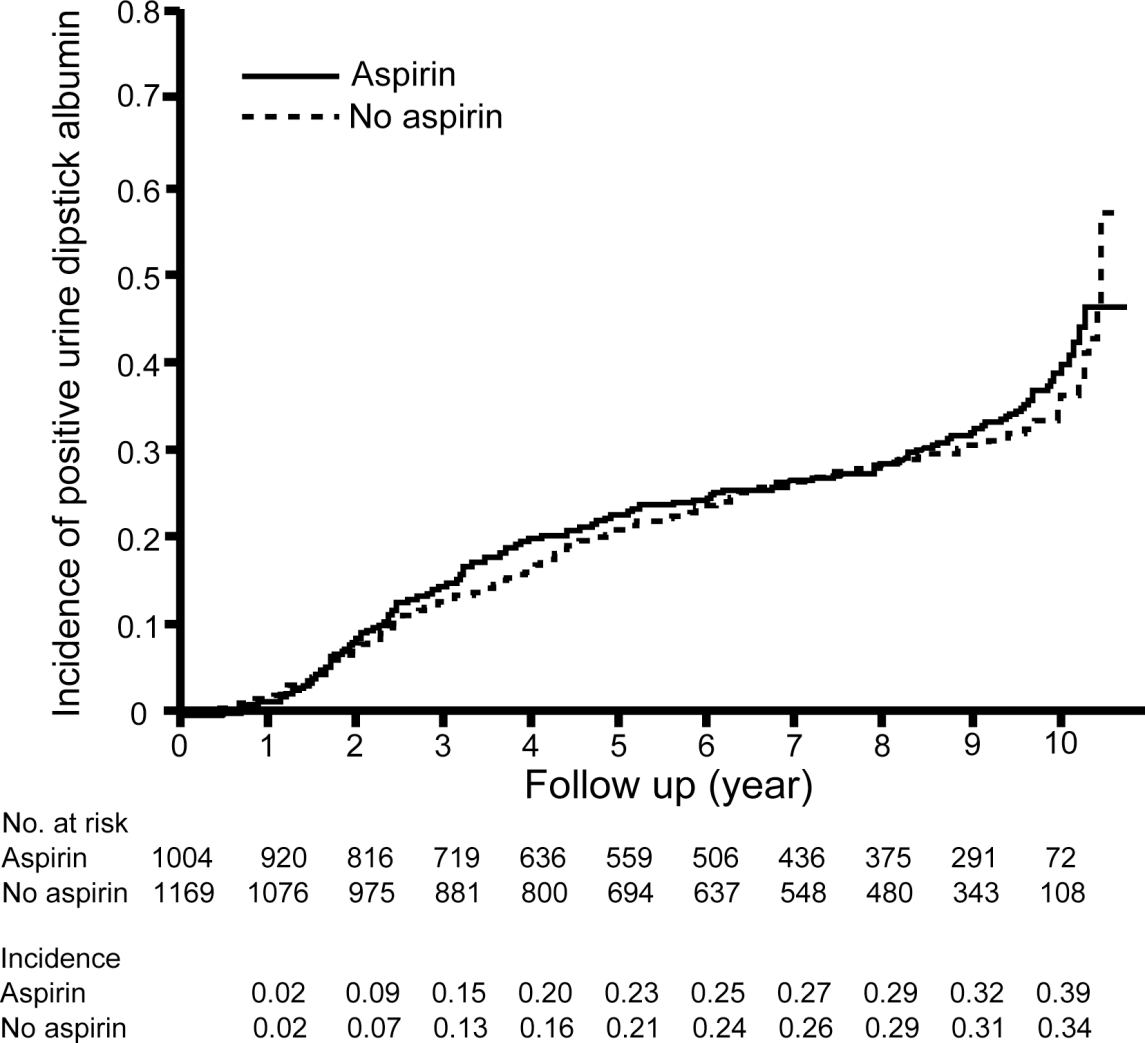
Incidence of positive urine dipstick albumin in patients on long-term low-dose aspirin therapy in the on-treatment analysis. In the on-treatment analysis, low-dose aspirin had no effect on the incidence of positive urine dipstick albumin (HR, 1.08; 95% CI, 0.92 to 1.28; log-rank *P* = 0.32).

Since the JPAD2 cohort study was a cohort study, the use of low-dose aspirin was based on each treating physician’s clinical judgment after the RCT period of the JPAD trial. In July 2013 survey, 881 (82%) patients in the aspirin group and 123 (11%) in the no aspirin group were taking low-dose aspirin (Fig 1B). When we took into account the actual use of low-dose aspirin (on-treatment analysis), the incidence of positive urine dipstick albumin was not associated with the use of low-dose aspirin (HR, 1.08; 95% CI, 0.92 to 1.28; log-rank *P* = 0.32; Fig 3).

### Multivariable analysis of the incidence of positive urine dipstick albumin

The multivariable Cox proportional hazards models were performed in 2,159 patients, since there were 14 missing data in serum creatinine at baseline. The multivariable analyses showed that low-dose aspirin was not associated with the incidence of positive urine dipstick albumin in the intension-to-treat analysis (HR, 1.12; 95% CI, 0.95 to 1.32) and the on-treatment analysis (HR, 1.08; 95% CI, 0.92 to 1.27) (Table 2). The incidence of positive urine dipstick albumin was higher among the elderly (≥65 years old) and those with elevated serum creatinine, high HbA1c (≥7.2%), or high blood pressure in both analyses.

**Table 2:** Multivariable analysis of the incidence of positive urine dipstick albumin.

	HR	95% CI		
Intention-to-treat analysis				
Aspirin use	1.12	0.95	to	1.32
Age ≥65 years	1.23	1.04	to	1.46
Male sex	0.95	0.79	to	1.15
Serum creatinine	2.59	1.71	to	3.93
HbA1c ≥7.2%	1.38	1.16	to	1.63
Systolic BP ≥140 mmHg or diastolic BP ≥90 mmHg	1.78	1.48	to	2.13
ACE inhibitor or ARB use at baseline	1.11	0.91	to	1.35
ACE inhibitor / ARB use at the time of the follow-up survey in 2009	0.98	0.81	to	1.18
On-treatment analysis				
Aspirin use	1.08	0.92	to	1.27
Age ≥65 years	1.24	1.04	to	1.46
Male sex	0.95	0.79	to	1.15
Serum creatinine	2.61	1.72	to	3.96
HbA1c ≥7.2%	1.38	1.16	to	1.63
Systolic BP ≥140 mmHg or diastolic BP ≥90 mmHg	1.80	1.50	to	2.15
ACE inhibitor or ARB use at baseline	1.10	0.91	to	1.33
ACE inhibitor / ARB use at the time of the follow-up survey in 2009	0.99	0.82	to	1.19

BP indicates blood pressure; ACE, angiotensin-converting enzyme; and ARB, angiotensin II type 1 receptor blocker.

### Annual changes in eGFR

The analysis of annual changes in eGFR included 1,574 patients (aspirin group, 766; no aspirin group, 808), because there were 599 missing data in eGFR at follow-up. The annual change in eGFR was −0.8 ± 2.9 ml/min/1.73m^2^/year in the aspirin group, and −0.9 ± 2.5 ml/min/1.73m^2^/year in the no aspirin group. There was no significant difference in the both group (*P* = 0.2).

## Discussion

Low-dose aspirin therapy is recommended for preventing CVD in high-risk patients with diabetes [14, 15]. However, it remained uncertain whether long-term low-dose aspirin use affects renal function in patients with diabetes. Also in people without diabetes, the association between aspirin and the risk of CKD was controversial.

Previous case-control studies that estimated the cumulative aspirin dose in patients with CKD and those without CKD have produced conflicting results. Some case-control studies have found a positive association between aspirin use and CKD prevalence [4, 5], while other studies have found no association [6, 7].

Several large cohort studies were conducted to examine whether long-term aspirin use affects renal function in healthy people and patients with CKD. One cohort study in healthy people was based on the Physicians’ Health Study. In this study, a total of 11,032 initially healthy men were followed for 14 years. The association between renal function based on serum creatinine and creatinine clearance and cumulative aspirin dose was not observed [8]. Another cohort study analyzed the association between changes in eGFR over 11 years and cumulative aspirin dose among 1,697 initially healthy women from the Nurses’ Health Study. The study concluded that long-term aspirin use was not associated with renal dysfunction [10]. On the other hand, a cohort study of 19,163 patients with newly diagnosed CKD in Taiwan found that aspirin use was associated with an increased risk of ESRD [11]. The effect of aspirin on renal dysfunction in healthy individuals and patients with CKD may differ.

Most clinical studies evaluated renal function using serum creatinine, creatinine clearance, or eGFR, not albuminuria nor proteinuria. Recently, a nationwide cross-sectional study based on data from the National Health and Nutrition Examination Survey analyzed the association between habitual analgesic use and albuminuria prevalence in 8,057 US adults [13]. It showed that the prevalence of albuminuria (urinary albumin-to-creatinine ratio of 30 mg/g or greater) was similar in people with habitual aspirin use and those without habitual use of any analgesics. Since albuminuria and proteinuria occur before eGFR declines in some conditions such as diabetes [17], this study was notable in using albuminuria as a marker of renal dysfunction. However, this study was a cross-sectional study. Prospective studies were needed to assess the effect of low-dose aspirin on albuminuria or proteinuria.

A previous prospective study reported ‘high-dose’ aspirin reduced proteinuria in patients with diabetes. This study showed that 6 weeks of aspirin (990 mg/day) and dipyridamole (225 mg/day) reduced urinary protein excretion without changes in blood pressure and plasma glucose levels in 16 patients with type 1 diabetes [23]. Although the sample size was small, the results suggested that short-term high-dose aspirin had beneficial effects on proteinuria in patients with diabetes. Subsequently, more prospective studies were conducted to examine whether ‘low-dose’ aspirin affects albuminuria in patients with diabetes. These studies recruited patients with diabetes and microalbuminuria (urinary albumin excretion of 30 to 300 mg/day), who were then treated with low-dose aspirin (150 mg/day) for 4 weeks. There were no changes in urinary albumin excretion and eGFR [24, 25], suggesting that low-dose aspirin therapy does not affect albuminuria in patients with diabetes. However, these studies had relatively short follow-up periods. Long-term studies were required to evaluate the effect of low-dose aspirin on albuminuria or proteinuria in patients with diabetes.

The present study assessed for the first time whether long-term low-dose aspirin affects the incidence of positive urine dipstick albumin in patients with type 2 diabetes. Both intention-to-treat analysis and on-treatment analysis showed that low-dose aspirin did not increase the incidence of positive urine dipstick albumin for a median follow-up of 8.5 years. The multivariable Cox proportional hazards models also showed that low-dose aspirin did not increase the risk of positive urine dipstick albumin, adjusting age, sex, serum creatinine, hemoglobin A1c, blood pressure, blood pressure, and treatment with ACE inhibitors and ARBs.

Our study has several limitations. First, we did not have data about habitual analgesic use. Habitual analgesic use might affect the relationship between low-dose aspirin and renal function. Second, we did not quantify the degree of albuminuria. It is possible that we underestimated the effect of aspirin on renal function, because albuminuria is a very early change in diabetic nephropathy. Third, we measured urine dipstick albumin, not by evaluating the urinary albumin–creatinine ratio. Dipstick-positivity may be affected by various conditions, such as sample volume and urine specific gravity. Finally, the JPAD2 cohort study was a cohort study after the RCT period. However, over 80% of patients remained in the original allocation for aspirin as of the 2013 follow-up. The on-treatment analysis and multivariable analysis also showed similar results.

We concluded that low-dose aspirin therapy does not affect eGFR and the incidence of positive urine dipstick albumin during a median follow-up of 8.5 years. Although aspirin is a type of cyclooxygenase inhibitor, our findings suggest that long-term low-dose aspirin therapy is safe with respect to renal function for patients with type 2 diabetes.
